# Supplementary material for: Emerging threat of ranavirus: prevalence, genetic diversity, and climatic drivers of Ranavirus (Iridoviridae) in ectothermic vertebrates of Asia
Source: Front Vet Sci. 2023 Nov 23;10:1291872. doi: 10.3389/fvets.2023.1291872 (PMC10701541; doi:10.3389/fvets.2023.1291872)
Supplement: Supplementary file 1 [file Data_Sheet_1.pdf]

Supplementary Material

Emerging Threat of Ranavirus: Prevalence, Genetic Diversity, and Climatic Drivers of *Ranavirus* (*Iridoviridae*) in Ectothermic Vertebrates of Asia

Jayampathi Herath, Dan Sun, Gajaba Ellepola, Kuttichantran Subramaniam, Madhava Meegaskumbura*

*** Correspondence:** Madhava Meegaskumbura: madhava_m@mac.com

1 Supplementary Data

1.1.1. Genetic supplementary data

Seventy-eight sequences representing Rana nigromaculata ranavirus and a single sequence of the tiger frog virus were deposited in the Genbank.

The following are the Genbank accession numbers for the deposited 78 sequences.

Rana nigromaculata ranavirus MCP gene fragment: OR487873, OR487874, OR487875, OR487876, OR487878, OR487879, OR684375-OR684445
Tiger frog MCP gene fragment: OR487877

2 Supplementary Figures and Tables

2.1 Supplementary Tables

Table S1: Model used for the analysis. This includes different possible combinations.

No	Model	K	AICc	DAICc	Weight	LogLiK
1	bio15	2	51.31	0	0.25	-23.16
2	bio18	2	51.94	0.62	0.18	-23.47
3	bio15+bio18	3	53.29	1.98	0.09	-22.55
4	bio08+bio15	3	53.8	2.48	0.07	-22.81
5	bio18+elevation	3	53.92	2.6	0.07	-22.87
6	bio15+elevation	3	54.3	3.1	0.05	-23.06
7	bio15+lifes stage	3	54.42	3.33	0.05	-23.12
8	bio18+life stage	3	54.64	3.33	0.05	-23.23
9	bio08	3	54.64	4.97	0.05	-25.64
10	bio07+bio15+bio18	2	56.29	5.34	0.02	-22.33
11	bio07+bio08+bio18	4	56.66	5.65	0.02	-22.48

12	bio15+bio18+life stage	4	56.97	5.8	0.01	-22.55
13	bio18+elevation+life stage	4	57.11	5.87	0.01	-22.59
14	bio08+bio15+life stage	4	57.18	6.13	0.01	-22.72
15	bio07+bio08+elevation	4	57.44	6.27	0.01	-22.79

Table S2: Bioclimatic variables

bio1 = Annual Mean Temperature
bio2 = Mean Diurnal Range (Mean of monthly (max temp - min temp))
bio3 = Isothermality (BIO2/BIO7) (×100)
bio4 = Temperature Seasonality (standard deviation ×100)
bio5 = Max Temperature of Warmest Month
bio6 = Min Temperature of Coldest Month
bio7 = Temperature Annual Range (BIO5-BIO6)
bio8 = Mean Temperature of Wettest Quarter
bio9 = Mean Temperature of Driest Quarter
bio10 = Mean Temperature of Warmest Quarter
bio11 = Mean Temperature of Coldest Quarter
bio12 = Annual Precipitation
bio13 = Precipitation of Wettest Month
bio14 = Precipitation of Driest Month
bio15 = Precipitation Seasonality (Coefficient of Variation)
bio16 = Precipitation of Wettest Quarter
bio17 = Precipitation of Driest Quarter
bio18 = Precipitation of Warmest Quarter
bio19 = Precipitation of Coldest Quarter

Table S3: Infected native species with their life stage and IUCN conservation status. Conservation status is based on IUCN categories with acronyms representing from low to high extinction risk: LC, least concern; NT, near threatened; VU, vulnerable; EN, endangered; DD, Data Deficiency

Family	Species	Infected life stage	IUCN status
Ranidae	*Amolops ricketti*	Adults	LC
	Amolops chunganensis	Adults	LC
	Odorrana gramine	Adults	LC
	Odorrana versabilis	Adults	LC
	Rana hanluica	Adults	LC
	Hylarana guentheri	Tadpoles	LC
Dicroglossidae	*Quasipaa spinosa*	Adults	VU
	Quasipaa boulengeri	Adults	VU
Hylidae	*Hyla simplex*	Tadpoles	LC
Microhylidae	*Microhyla pulchra*	Tadpoles	LC
Rhacophoridae	*Polypedates megacephalus*	Tadpoles	LC
Cobitidae	*Paramisgurnus dabryanus*	Adults	-
Channidae	*Channa argus*	Adults	LC
Geoemydidae	*Mauremys mutica*	Adults	CR

2.1. Supplementary Figures

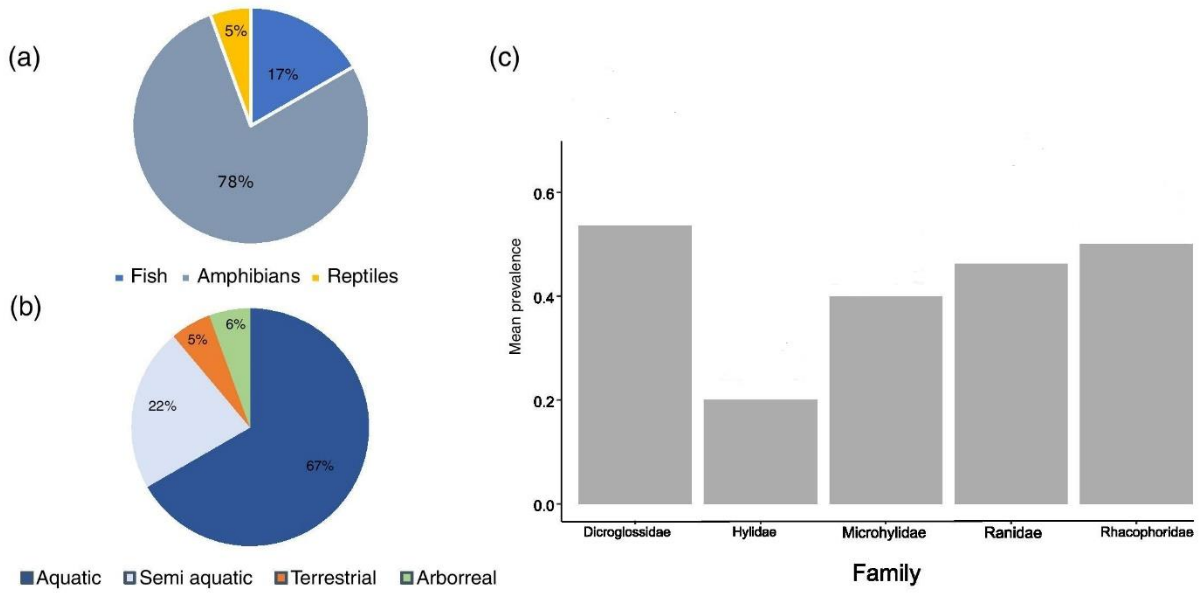

Figure S1: Epidemiological characteristics of the disease. (a) Number of species infected according to class. (b) Number of species infected according to their habitat. (c) Mean infection prevalence percentages among different families of anurans. Ranidae (n = 7 species), Dicroglossidae (n = 3 species), Rhacophoridae (n = 1 species), Microhylidae (n = 1 species), Hylidae (n = 1 species).

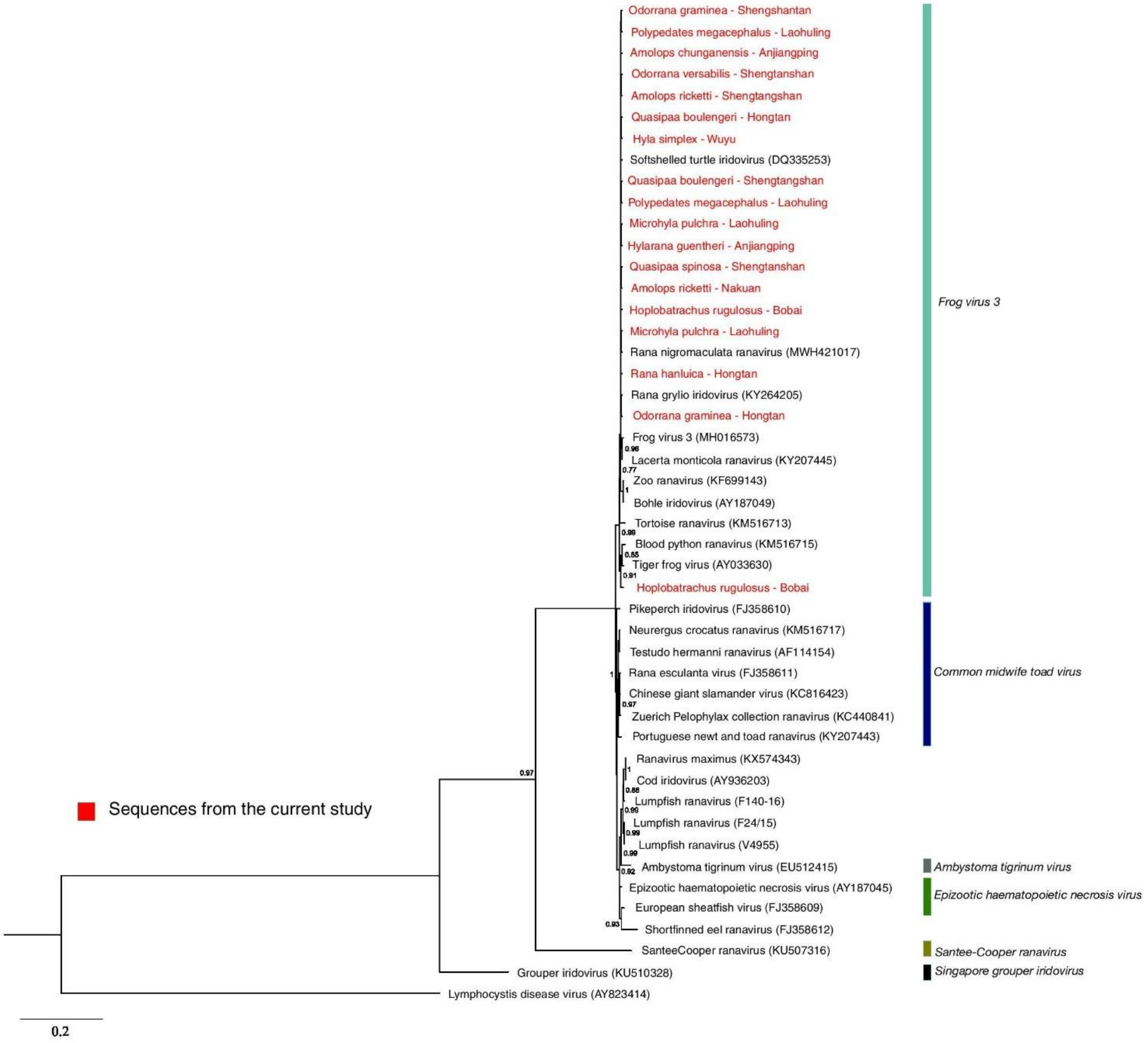

Figure S2: Phylogenetic tree based on the MCP gene sequences by BEAST v.2.7.4

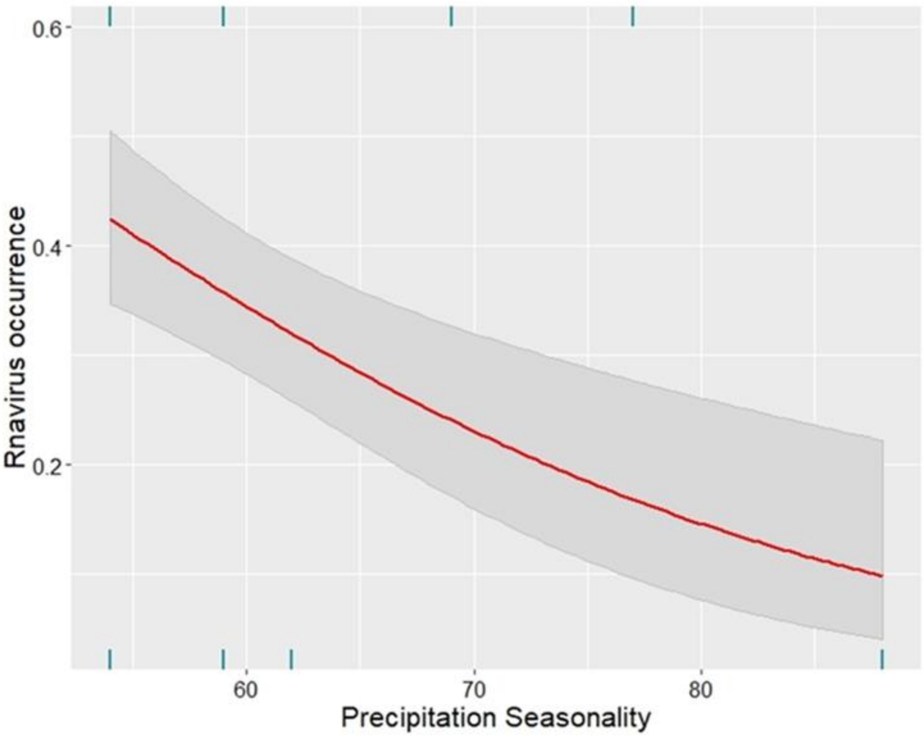

Figure S3: Precipitation seasonality.

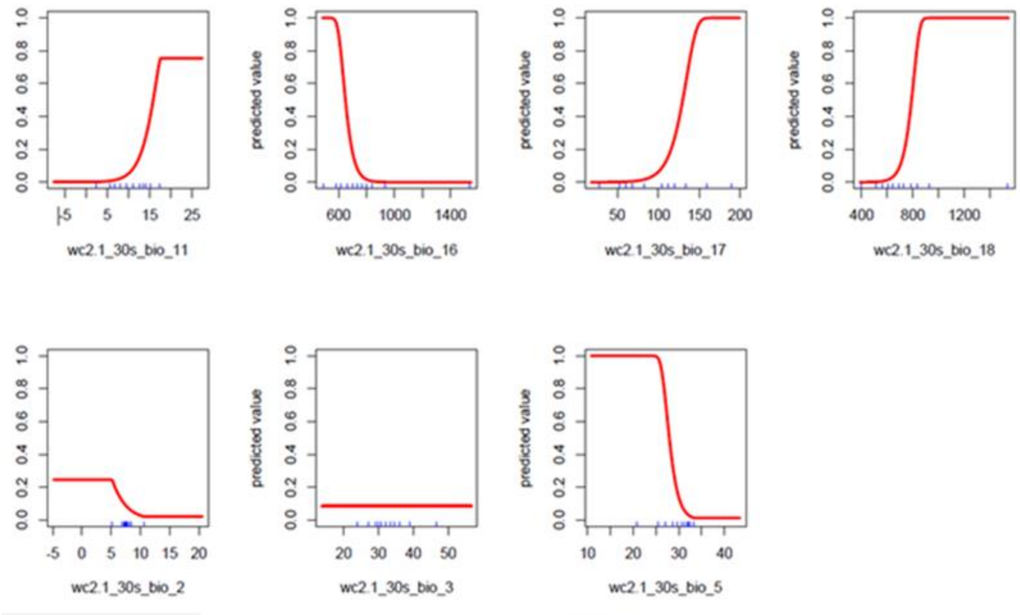

Figure S4: Response curves.
